# Twenty-Four-Hour Real-Time Continuous Monitoring of Cerebral Edema in Rabbits Based on a Noninvasive and Noncontact System of Magnetic Induction

**DOI:** 10.3390/s17030537

**Published:** 2017-03-08

**Authors:** Gen Li, Ke Ma, Jian Sun, Gui Jin, Mingxin Qin, Hua Feng

**Affiliations:** 1 College of Biomedical Engineering, Third Military Medical University, Chongqing 400038, China; calvin_gen@163.com (G.L.); ywqmk@sohu.com (K.M.); tjjingui@126.com (G.J.); 2 College of Bioengineering, Chongqing University, Chongqing 400030, China; 3 Department of Neurosurgery, Southwest Hospital, Chongqing 400038, China; fenghua_swh@sina.com

**Keywords:** cerebral edema, noncontact, magnetic induction, intracranial pressure, real-time monitoring

## Abstract

Cerebral edema is a common disease, secondary to craniocerebral injury, and real-time continuous monitoring of cerebral edema is crucial for treating patients after traumatic brain injury. This work established a noninvasive and noncontact system by monitoring the magnetic induction phase shift (MIPS) which is associated with brain tissue conductivity. Sixteen rabbits (experimental group *n* = 10, control group, *n* = 6) were used to perform a 24 h MIPS and intracranial pressure (ICP) simultaneously monitored experimental study. For the experimental group, after the establishment of epidural freeze-induced cerebral edema models, the MIPS presented a downward trend within 24 h, with a change magnitude of −13.1121 ± 2.3953°; the ICP presented an upward trend within 24 h, with a change magnitude of 12–41 mmHg. The ICP was negatively correlated with the MIPS. In the control group, the MIPS change amplitude was −0.87795 ± 1.5146 without obvious changes; the ICP fluctuated only slightly at the initial value of 12 mmHg. MIPS had a more sensitive performance than ICP in the early stage of cerebral edema. These results showed that this system is basically capable of monitoring gradual increases in the cerebral edema solution volume. To some extent, the MIPS has the potential to reflect the ICP changes.

## 1. Introduction

Cerebral edema is a common secondary disease following traumatic brain injury (TBI) which can be an important risk factor for mortality and poor outcome [1,2]. Therefore, the real-time continuous monitoring of cerebral edema plays important roles in disease observation, treatment guidance, surgery timing determination and prognosis evaluation for patients after TBI. Cytotoxic (cellular) and vasogenic edema are two major types of edema. Both types of edema can develop into a vicious cycle until the brain swells uncontrollably, resulting in intracranial pressure (ICP) and permanent brain damage [3]. Cytotoxic cerebral edema is most commonly observed after TBI and can include cell swelling and the intracellular accumulation of water. The blood-brain barrier (BBB) is disrupted and can cause extracellular edema in vasogenic cerebral edema [4]. The development of cerebral edema can often be observed in three ways: rapid development (within 24–36 h), gradually progressive development (over several days), or initially worsening development (about a week) [5]. There are many invasive and noninvasive techniques for edema monitoring. In 2014, the American Heart Association/American Stroke Association (AHA/ASA) recommended that it is useful to measure computerized tomography (CT) and magnetic resonance imaging (MRI) images within 6 h of a traumatic brain injury. Serial CT findings and ICP monitoring in the first twodays are useful to identify patients at high risk for developing symptomatic swelling [5]. Transcranial doppler (TCD) sonography could also be used as a noninvasive method for edema assessment. Invasive techniques are associated with complication risks, such as hemorrhage and infection [6]. Furthermore, no effective method has been found to reliably predict the development of brain swelling. Noninvasive techniques such as CT/MRI have no risk of complication. However, CT/MRI cannot be applied for real-time monitoring and relies on the doctor’s individual choices, which may delay the diagnosis. Other noninvasive methods such as TCD cannot monitor edema accurately enough to be routine alternatives to invasive measurements. The electrical bioimpedance (EBI) can reflect the physical properties and variation of intracranial lesions by testing the electric potential of the surface of the skull and its changes in order to monitor the whole process of occurrence, development and healing of cerebral edema [7]. However, in practice, problems such as the injection current decay and poor penetration caused by resistance contacting the electrode epidermis and the high resistivity of the skull seriously affect the measurement accuracy. Therefore, clinical practice needs a method for bedside and real-time monitoring of edema development.

The magnetic induction phase shift (MIPS) method acts an alternating main magnetic field (B) on the object to produce an induced eddy current which forms a disturbing magnetic field (ΔB) [8]. Electrode-less measurement of the changing conductivity in the human body can be achieved by measuring the effect of inducted eddy currents [9]. Different biological tissues have different electromagnetic properties and therefore can be differentiated on the basis of these features [10]. Currently this method has been applied to study the moisture content of the brain, brain hematoma, dielectric constant and cardiopulmonary activities [11,12,13,14,15,16]. Therefore, we assume that measuring the change in the MIPS could be a noncontact method for the bedside monitoring of cerebral edema to show its development. However, the conductivity of the biological tissue is very small (σ < 3 s/m), and the magnetic field disturbance produced by the inducted eddy currents is very weak [10]. Moreover, the target field accounts for a small proportion of the whole disturbing magnetic field [13]. Hence, the signal of interest is extremely weak. Therefore, our previous works were devoted to increasing the measurement sensitivity. In 2014, we established a magnetic induction phase detection system, which could achieve phase noise as low as 6 m° and a 4h phase drift as low as 30 m° at 21.4 MHz [17] and studied the MIPS of cerebral hemorrhage in rabbits with a single coil-coil [18]. Furthermore, we designed a symmetric cancellation-type sensor detection system based on the symmetry between the two brain hemispheres so as to offset the interference of the primary field and the perturbation field generated by other brain tissues [19]. Although the sensitivity was somewhat improved, the desired result was not achieved. The volumetric induction phase-shift spectroscopy (VIPS) method presented by Gonzalez et al. [20] is self-referencing and can be performed in a short time, providing instantaneous information. As reported in 2013, VIPS data can be used for diagnosis to detect brain damage and even distinguish patients with edema from those with hematoma [21]. However, the excitation signals used by a single or a limited number of frequencies were restricted to learn detailed information about intracranial lesions from large amounts of MIPS data measured on a wide frequency band and to accurately find the frequency with optimal sensitivity.

In this study, a magnetic induction brain monitor was designed to sweep the measured target within the entire frequency range of 300 kHz–200 MHz and to obtain the detailed MIPS data in the frequency range. Such a magnetic induction brain monitor can finish excitation signal output, phase detection, and data acquisition automatically. Furthermore, the real-time MIPS change trend at an optimal sensitive frequency can be observed continuously by using it. As a gold standard for monitoring cerebral edema, ICP can reflect intracranial brain edema through indirect measurement [3]. To demonstrate that the MIPS method can monitor the cerebral edema in real time and offer a new potential method for clinical application, we designed a 24 h simultaneous MIPS and ICP monitoring experiment in rabbits.

## 2. Materials and Methods

### 2.1. MIPS Detection Principle

MIPS detection technology is a one-dimensional magnetic induction tomography (MIT) technology that is applied to detect biological tissues [8,20]; it has been utilized by scholars globally. Its principle is to use a sinusoidal excitation signal with a certain frequency to act on the excitation coil and thereby generates an alternating main magnetic field (B) that acts on the object to produce an induced current; the induced current forms a disturbing magnetic field (ΔB). The detection coil receives the superimposed magnetic field (B + ΔB) of the main magnetic field (B) and the disturbing magnetic field (ΔB); the superimposed magnetic phase shift occurs with respect to the main magnetic field phase [9].The phase shift (φ) during this process is the MIPS. The MIPS is in proportion to the conductivity of the object and the frequency of the excitation signal [22].

### 2.2. Construction of the System

**Figure 1:**
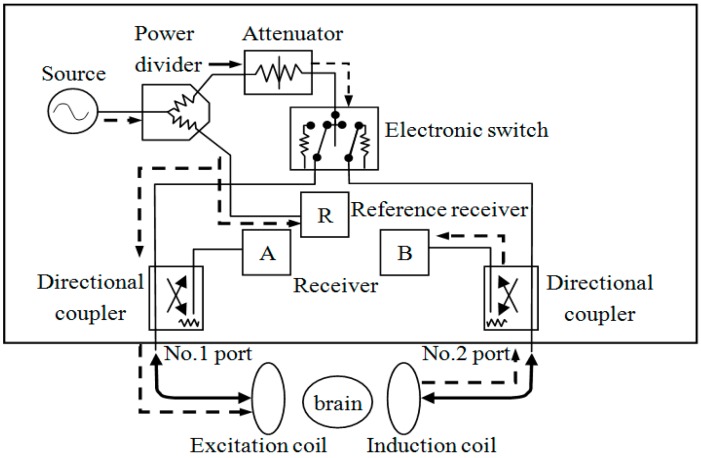
The equivalent circuit of the noninvasive and noncontact system of magnetic induction.

This system is mainly composed of two modules: the self-designed magnetic induction brain monitor (CNJY-2015, Third Military Medical University, Chongqing, China) and a two-coil sensor. The equivalent circuit of the system was shown in Figure 1.

#### 2.2.1. Two-Coil Sensor

**Figure 2:**
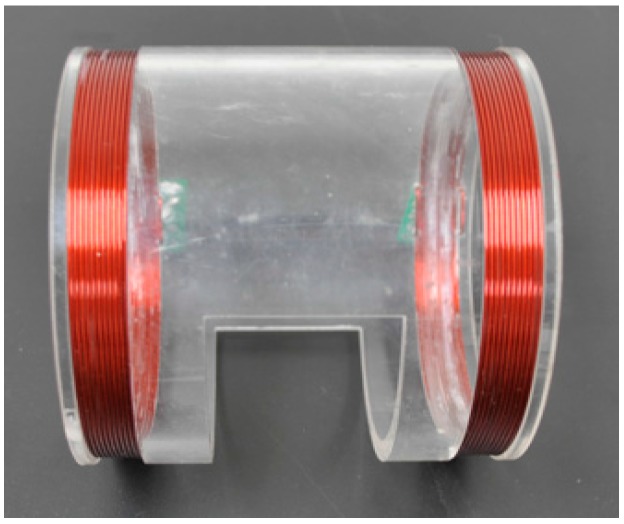
The two-coil sensor for rabbits experiment.

The sensor coil is an important part of MIPS detection system. In previous works, we intensively studied on the relationship between the coil structure and MIPS measurement and designed symmetric cancellation-type coil, coaxial coil, double-end exciting coil and helmholtz coil, the result of which showed that the coil with different structure had different sensitive areas and that the coil with the same axis could generate heterogeneous magnetic energy [18,19]. In addition, previous studies on biological tissue magnetic induction tomography (MIT) were based on the traditional theory of single exciting coil–single induction coil [8,9,10,14]. For general applicability and value of research, this study chose two coils with the same axis in parallel, as shown in Figure 2, both of which were circled by copper lines at the two ends of organic glass pipe with a diameter of 1 mm, one as excitation coil, another as detection coil, and the number of turns N1 = N2 = 10 [12,13,19]. In order to conveniently place rabbits in animal experiment and make the size of coils suitable for the heads of rabbits, excitation coil and detection coil were designed with the same radius (R1 = R2 = 5.2 cm) and with a distance of 10 cm. The rabbit head was placed in the lower middle of the two coils, and the two coils were connected to two ports of the magnetic induction brain monitors with high frequency coaxial wire. Moreover, at the suitable position of the organic glass pipe, a hole was drilled large enough for researchers to use the Camino MPM-1 intracranial pressure monitor in animal experiment.

#### 2.2.2. Design of the Magnetic Induction Brain Monitor

The magnetic induction brain monitor was designed with functions of excitation signal output, phase detection, data acquisition and real-time display. The source can output two channels of sinusoid signals with a common frequency band and consistent initial phase, one of which was exerted on the main magnetic field through the excitation coil. The main magnetic field caused the brain tissue to induce current to form the disturbance magnetic field, and the detection coil received the sum of the main magnetic field and the disturbance magnetic field. The other signal was taken as a reference signal. At this time, MIPS was produced by subtracting the phase of the detection coil signal (θ_det_) from the phase of the reference signal (θ_ref_). Its formula was as follows:

MIPS = θ_det_ − θ_ref_ (1)

The magnetic induction brain monitor also contains the software to control the whole system that can help realize the automatic setting of the measurement parameters. The starting and termination frequencies of the excitation source scanning were set to 300 kHz and 200 MHz. The scanning points were 1601, and the scanning power was 10 dbm. The trigger source was an internal source, and the trigger mode was continuous. After that, 1601 MIPS data in the frequency range of 300 kHz–200 MHZ were obtained. The software has a monitoring interface for cerebral edema that allows researchers to possess the ability of setting the detection frequency. In addition to the MIPS data obtained in the frequency range of 300 kHz to 200 MHz, a magnetic induction amplitude in the same frequency range was obtained. The greater the amplitude of the frequency, the higher the MIPS detection sensitivity and the better the consistency among samples [15,16,20].Therefore, the software automatically defaults the detection frequency to the frequency with the highest amplitude. As the trigger mode of magnetic induction brain monitor was continuous, the MIPS of the detection frequency was produced continuously. Then, the software read the MIPS continuously at a rate of 60 times/h. Subsequently, the real-time MIPS trend of the detection frequency can be continuously displayed on the monitoring interface.

### 2.3. Animal Experimental Design

All animal experiments were performed in accordance with the guidelines from the Administration of Animal Experiments for Medical Research Purposes issued by the Ministry of Health of China. The protocol used was reviewed and approved by the Animal Experiments and Ethical Committee of Third Military Medical University (TMMU, Chongqing, China). All efforts were made to minimize the suffering of rabbits during experiments. Sixteen rabbits (available from Daping Hospital, weighing 2.0–2.6 kg, marked with No. 1 to No. 16) were randomly selected and divided into an experimental group (*n* = 10) and a control group (*n* = 6).

**Figure 3:**
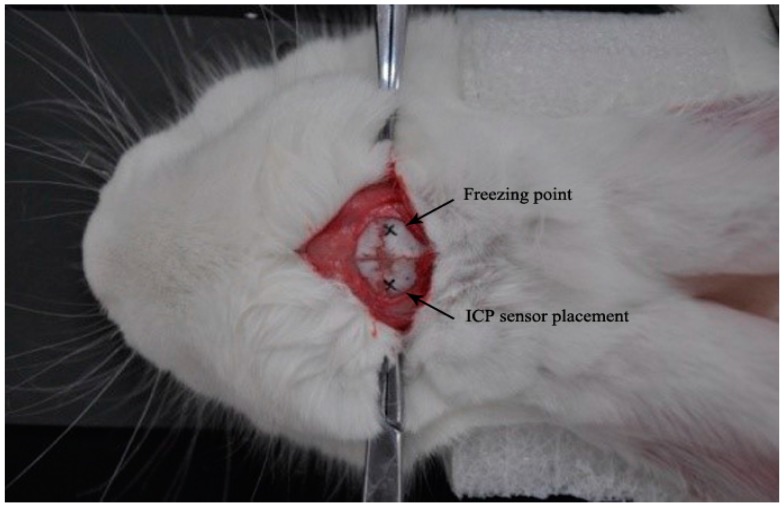
Rabbit freezing point and ICP sensor placement.

The epidural freezing method that can lead to cerebral edema rapidly [23] was used to establish a cerebral edema animal model that was fixed to the platform of the table by non-magnetic material. The mechanism of the frozen traumatic brain injury model was single, simple, and easy for qualitative research [24]. In the experimental group, rabbits were first injected with urethane (25%, 5 mL) by an ear vein for anesthesia. The hair was removed from the head and then a hole was drilled beside the “cross stitch” of the brain, forming a 5 mm × 5 mm bone window and exposing the dura mater; then, the cryopencil was immersed in liquid nitrogen for a sufficient time and was put into the bone window for freezing. The freezing temperature was −196 °C, the freezing time was 120 s, and the dental cement was used to seal the bone window. Through the experiment, ICP was taken as the reference signal. To embed the fiber optic probe of the Camino MPM-1 intracranial pressure monitor, a hole with a diameter of 2 mm was re-opened in animal models, and the opening and freezing points were axially symmetrized by a sagittal suture. Then, the optical fiber probe was inserted approximately 7 mm into the brain parenchyma and was closed and fixed with gudgeons. The rabbits in the control group experienced the same procedure only without freezing. The freezing point and ICP sensor placement are shown in Figure 3.

**Figure 4:**
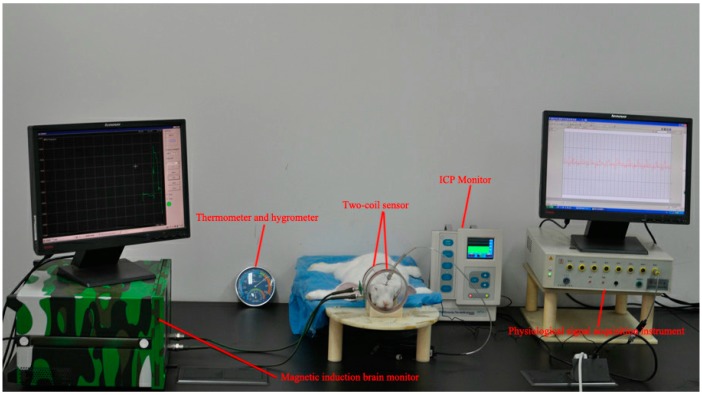
Experimental setup to monitor cerebral edema in rabbits with the MIPS method.

As shown in Figure 4, each animal was placed into the coils after the operation. A thermometer and hygrometer displayed the temperature and humidity at real time, and the temperature of the experiment environment was controlled to 21–23 °C, while humidity was maintained at approximately 50%. A physiological signal acquisition instrument (RM6280C, Chengdu Instrument Factory, Chengdu, China) was used for continuously monitoring the heart rate changes in the rabbits. When the heart rate of the experimental animals was regular, a magnetic induction brain monitor and Camino MPM-1 intracranial pressure monitor were used to monitor the 24 h MIPS trends and ICP changes at real time. During the monitoring process, rabbits were intraperitoneally injected with 5 mL of 25% urethane every 2 h; vital signs were monitored to ensure stability.

### 2.4. MIPS and ICP Data Analysis

The initial sampling rate of the MIPS signal was 60 times/h. Because the original signal was mixed with heart and lung activities, thus a wavelet transform was used to reduce noise and filter and then re-sampled the signal at a rate of 1 times/h. Moreover, the initial phase is set as zero, which can enable the more intuitive observation of the change in the MIPS absolute value. The initial sampling rate of ICP was 1000 Hz; because of the huge amount of data, the processing software of the physiological signal acquisition instrument was first pre-treated. The auto interval of signal was 300 s. The data from the last 60 s of each 300 s were extracted; the mean sequence was obtained, and then the data were re-sampled at a rate of 1 times/h. Eventually the MIPS and ICP monitoring results of the 16 rabbits were averaged.

### 2.5. Brain Water Content Analysis

Cerebral edema is defined as an abnormal accumulation of fluid associated with volumetric enlargement of the brain. Therefore, brain water content (BWC), which reflects increasing water content, is widely used to evaluate cerebral edema in experimental studies [25]. After 24 h MIPS and ICP monitoring experiment, brain wet weights (BWW) were measured in right hemispheres (freezing side) of the experimental rabbits and right hemispheres (control side) of the control rabbits after removal of cerebellum, pons, and olfactory bulbs. Dry weights (DW) were determined after 72 h heating at 100°C, and percentage BWC in each sample was calculated as [(BWW−DW)/WW] × 100% [26].

### 2.6. Statistical Analysis

All of the data are expressed as the mean ± standard deviation from six independent experiments at least. The Levene F was used to test the equal variances assumed of two samples. One-way ANOVA was used to determine whether there is a significant difference in the average deviation. The MIPS and ICP data were analyzed with a bilaterally independent *t*-test and simple Pearson correlation coefficients. The BWC data were analyzed with a bilaterally independent *t*-test. Statistical analyses were performed by SPSS software version 19.0 (SPSS Inc., Chicago, IL, USA). The significance level was set at *p* < 0.05.

## 3. Results

**Figure 5:**
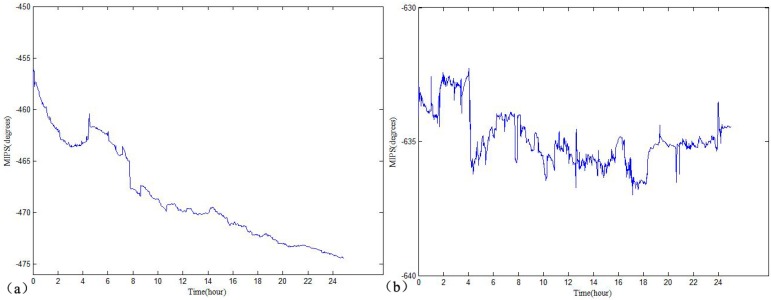
(**a**) The 24 h MIPS results of the No. 3 rabbit in the experiment group; (**b**) 24 h MIPS results of the No. 12 rabbit in the control group.

The MIPS results of the No. 3 and No. 12 rabbits are respectively shown in Figure 5a,b. For the No. 3 rabbit that received the freezing treatment, the MIPS overall declined over time during the 24 h monitoring process. For the No. 12 rabbit that did not receive the freezing treatment, the MIPS also changed over time, but there was no clear upward or downward trend during the 24 h monitoring process.

**Figure 6:**
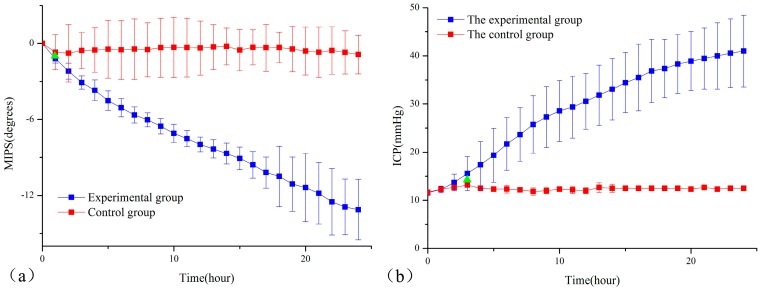
The mean ± standard deviation 24 h trends of MIPS and ICP. The green triangle indicated the time of a significant difference between the experimental group and control occurred. (**a**) Comparison of MIPS in experimental group and control group rabbit; (**b**) Comparison of ICP in experimental group and control group rabbit.

Figure 6a,b represent the mean 24 h trends of the MIPS and ICP of the experimental group (*n* = 10) and the control group (*n* = 6), respectively. For the experimental group, the mean MIPS showed a downward trend, while the mean ICP increased continuously. At the 24th hour, the mean ICP and MIPS had the largest change of 41 ± 7.44807 mmHg and −13.1121 ± 2.3953°, respectively. The mean MIPS of the control group had a change of −0.87795 ± 1.5146 degrees at the 24th hour but there was no clear upward or downward trend. The mean ICP of the control group showed a fluctuating trend at 12 mmHg. From this comparative analysis, it agrees with the theory that the occurrence of cerebral edema causes a decline in the electrical conductivity of brains, resulting in MIPS decline [20,27]. As a gold standard for monitoring cerebral edema, the ICP can reflect intracranial cerebral edema through indirect measurement [3]. The overall upward trend of the ICP in the experimental group indicated that the cerebral edema became increasingly severe within 24 h after the occurrence of cerebral edema. The 24th hour BWC results, as shown in Table 1, were analyzed with bilaterally independent *t*-tests, and the *p*-values were less than 0.05, indicating significant differences between the experimental and control groups. This can demonstrate that rabbits in the experimental group indeed had severe cerebral edema at the 24th hour, while rabbits in the control group almost did not have cerebral edema. The MIPS trend in this work is basically capable of monitoring gradual increases in the cerebral edema solution volume.

The results of the bilaterally independent *t*-test of the MIPS and ICP in the experimental and control groups are shown in Table 2. The *p*-value is 0.531 which is greater than 0.05 and the 95% confidence interval of the difference crossed zero, indicating no significant differences in the ICP between the experimental group and the control group at the initial time. Furthermore, it was observed that the ICP and MIPS began to show a significant difference at the third hour and the first hour, respectively, marked as green triangles in Figure 5a,b. In the early stage of increased ICP induced by cerebral edema, cerebrospinal fluid (CSF) with the maximal electrical conductivity tissue of the intracranial region played a leading role in regulation [28]. When cerebral edema occurred, the distribution of the cerebrospinal fluid immediately changed [2]. As part of CSF regulation, the ICP had a significant difference later than the MIPS. This result demonstrated that the MIPS was more sensitive than the ICP in the early stage of cerebral edema.

The MIPS monitors cerebral edema by monitoring the changes in the brain conductivity, and the ICP monitors cerebral edema by monitoring intracranial pressure changes caused by the development of cerebral edema. This study used simple Pearson correlation coefficients to statistically analyze the ICP and MIPS. The results in Table 3 show that the correlation coefficients of the MIPS and ICP of seven rabbits in the experimental group were between −0.7645 and −0.9153, and the *p*-values were all less than 0.05, indicating that the MIPS was negatively correlated with the ICP. Combined with the work of Sun [11,12], when both cerebral hemorrhage and cerebral edema lead to increased intracranial pressure, the ICP and MIPS are negatively correlated, further proving that the MIPS method is capable of reflecting the ICP induced by intracranial lesions.

## 4. Discussion

The MIPS detection method has the advantages of noninvasiveness, noncontact and strong penetration, overcoming the effect of the electrode-skin contact resistance of the electrical impedance methods. Moreover, the magnetic field can pass through the skull with high resistivity; hence, the MIPS method is superior in aspects such as detecting cerebral edema, cerebral hemorrhage, and cerebral ischemia [7,9].

Compared with previous detection systems that used single or limited numbers of frequencies [15,16,17,18,19,20,21], the system for cerebral edema established in this study can sweep within the entire frequency range (300 kHz–200 MHz) and can continuously observe the MIPS changes at an optimal sensitive frequency in real time with the monitoring software, improving the detection sensitivity greatly and realizing real-time continuous monitoring. The time for single MIPS collection was 0.628 s; this can fully meet the needs of 24 h real-time continuous monitoring for cerebral edema. The cerebral edema two-coil sensor is different from the cerebral hemorrhage coil sensor used by Jin [17], but both of them obtain a similar MIPS trend because cerebral hemorrhage is often associated with cerebral edema [29]. This structure of the two-coil sensor may not be able to distinguish between cerebral edema and cerebral hemorrhage.

The freezing-induced cerebral edema was mainly vasogenic cerebral edema accompanied by cytotoxic cerebral edema [30]. The BWC results illustrated that the rabbits in the experimental group appeared to have quite severe cerebral edema at the 24th hour after freezing, which agrees with the mechanism of the epidural frozen TBI model [24,31]. Currently, the MIPS method is not able to distinguish between these two types of cerebral edema. For rabbits in the experimental group, the MIPS decreased with the development of the cerebral edema, and the average maximum MIPS change at the 24th hour was −13.1121 ± 2.3953. However, the experimental data had some differences, primarily because of the presence of individual differences among the rabbits. Additionally, the original MIPS signal had glitches because of the differences in the depth of anesthesia for each rabbit and because of different levels of body movement in the 24 h monitoring process. The MIPS of rabbits in the control group also had changes because invasive ICP monitoring could induce slight intracranial hemorrhage within the 24 h monitoring process, thus leading to MIPS changes.

The ICP value of the rabbits in the experimental group did not increase rapidly; this is consistent with the compensatory mechanism of cerebral edema ICP monitoring. After the ICP increasesin the early stage of cerebral edema, it can be regulated through cerebrospinal fluid compensation and brain blood compensation within a certain range [3].When the compensatory capacity is exhausted, the ICP continues to increase [32]. The individual differences in rabbits led to different ICP compensatory abilities [27], and the ICP of some rabbits was always significantly increased, which may be the result of the poor compensation ability of ICP, resulting in edema exhaustion in the early stage, thus losing the ability to regulate the ICP. However, some rabbits have strong ICP compensatory abilities and can maintain a certain compensatory ability in the early edema stage. With aggravated edema, the ICP was significantly increased until the compensatory capacity was exhausted.

Cerebral edema in the early stage cannot be detected easily, which can also bring some harm [5]. As for the delay in ICP monitoring, the early sensitivity of the MIPS will be very helpful in the clinic, and it can improve the level of treatment by detecting cerebral edema with potential risks and helping make the decision for earlier intervention treatment. However, MIPS can be influenced by changes of the brain tissue temperature. The temperature after freezing for 1 h had a slight fluctuation compared with that before freezing. It indicates that the change of the MIPS for the first hour in the experimental group contained a small part of the temperature change. Therefore, in order to have a further study to show that the MIPS has higher sensitivity than ICP in the early stage of cerebral edema, it is necessary to eliminate the influence of temperature as much as possible.

## 5. Conclusions

This study establishes a new monitoring method for cerebral edema based on MIPS technology. This method has advantages of noninvasiveness, noncontact, good portability, and real-time continuous bedside monitoring. The simultaneous monitoring test results of cerebral edema MIPS and ICP of rabbits within 24 h and the BWC results show that the MIPS approach can be used to monitor gradual increases in the cerebral edema solution volume. In addition, the MIPS is more sensitive than the ICP in early stage of cerebral edema. To some extent, the MIPS has the potential to reflect ICP changes, as they are negatively correlated.

However, this is only a preliminary study, and our research team will conduct further studies to improve the effectiveness and reliability of the MIPS-based approach for monitoring cerebral edema, and to provide a new method for cerebral edema monitoring in clinical practice. Additionally, a real-time filtering method will be used to eliminate signal interference caused by body movement to improve the real-time monitoring results. Moreover, the middle cerebral artery occlusion method [33] will be used to establish a cerebral edema model compared with the existing freezing models. Cerebral edema monitoring in the clinic is more concerned with the changes of the cerebral edema solution volume [34,35]. Therefore, the relationship between changes of the MIPS absolute value and the changes of the cerebral edema solution volume should be found in the future. Under the condition of enough sample sizes, the MIPS measurement result with no edema can be a reference point (zero degree phase shift) of cerebral edema monitoring in real time. The detailed relationship between the MIPS and the cerebral edema solution volume will be quantitatively analyzed with the imaging method [36]. After that, the patient who already has cerebral edema can be diagnosed by real-time continuous monitoring of the changes of the cerebral edema solution volume.

## Figures and Tables

**Table 1:** BWC results from the experimental rabbits and the control rabbits at the 24th hour.

Sample	BWC (%)	
	Mean	Standard Deviation
experimental group	81.59	0.88
control group	78.03	0.51

**Table 2:** Independent *t*-test of ICP and MIPS from experiment group and control group.

Time	Sample	Variances	F	Significance	t	Significance (Two-Tailed)	95% Confidence Interval of the Difference	
							Lower	Upper
0 h	ICP1	Equal variances assumed	1.333	0.265	−0.641	0.531	−0.718	0.385
		Equal variances not assumed			−0.643	0.534	−0.743	0.403
1 h	MIPS1	Equal variances assumed	3.087	0.098	8.720	1.78 × 10^−7^	1.325	2.176
		Equal variances not assumed			7.490	1.29 × 10^−4^	1.199	2.302
1 h	ICP2	Equal variances assumed	0.029	0.868	−0.596	0.559	−0.759	0.426
		Equal variances not assumed			−0.620	0.548	−0.757	0.423
2 h	ICP3	Equal variances assumed	0.005	0.945	0.800	0.435	−0.412	0.912
		Equal variances not assumed			0.875	0.398	−0.368	0.868
3 h	ICP4	Equal variances assumed	0.754	0.398	2.502	0.024	0.115	1.385
		Equal variances not assumed			2.941	0.01	0.207	1.293

**Table 3:** Correlation analysis of ICP and MIPS from the experimental group.

Sample	Correlation Coefficient	*p* Value
No. 1 rabbit	−0.8742	0.01
No. 2 rabbit	−0.8208	0.01
No. 3 rabbit	−0.7645	0.01
No. 4 rabbit	−0.7865	0.01
No. 5 rabbit	−0.8674	0.01
No. 6 rabbit	−0.8857	0.01
No. 7 rabbit	−0.7804	0.01
No. 8 rabbit	−0.9153	0.01
No. 9 rabbit	−0.9092	0.01
No. 10 rabbit	−0.8027	0.01
